# A Powerful Mitochondria-Targeted Iron Chelator Affords High Photoprotection against Solar Ultraviolet A Radiation

**DOI:** 10.1016/j.jid.2016.03.041

**Published:** 2016-08

**Authors:** Olivier Reelfs, Vincenzo Abbate, Robert C. Hider, Charareh Pourzand

**Affiliations:** 1 Department of Pharmacy and Pharmacology, University of Bath, Claverton Down, Bath, UK; 2 Institute of Pharmaceutical Science, King’s College London, Franklin-Wilkins Building, London, UK

**Keywords:** ATP, adenosine triphosphate, LI, labile iron, LIP, labile iron pool, ROS, reactive oxygen species, TMRM, tetramethylrhodamine methyl ester, UVA, ultraviolet A

## Abstract

Mitochondria are the principal destination for labile iron, making these organelles particularly susceptible to oxidative damage on exposure to ultraviolet A (UVA, 320–400 nm), the oxidizing component of sunlight. The labile iron-mediated oxidative damage caused by UVA to mitochondria leads to necrotic cell death via adenosine triphosphate depletion. Therefore, targeted removal of mitochondrial labile iron via highly specific tools from these organelles may be an effective approach to protect the skin cells against the harmful effects of UVA. In this work, we designed a mitochondria-targeted hexadentate (tricatechol-based) iron chelator linked to mitochondria-homing SS-like peptides. The photoprotective potential of this compound against UVA-induced oxidative damage and cell death was evaluated in cultured primary skin fibroblasts. Our results show that this compound provides unprecedented protection against UVA-induced mitochondrial damage, adenosine triphosphate depletion, and the ensuing necrotic cell death in skin fibroblasts, and this effect is fully related to its potent iron-chelating property in the organelle. This mitochondria-targeted iron chelator has therefore promising potential for skin photoprotection against the deleterious effects of the UVA component of sunlight.

## Introduction

The ultraviolet A (UVA, 320–400 nm) component of sunlight is weakly absorbed by most biomolecules but is predominantly oxidative in nature, generating reactive oxygen species (ROS) via photochemical interactions involving non-DNA cellular chromophores (Tyrrell, 1996). In addition, the presence of a cellular pool of iron bound to small ligands, called labile iron pool (LIP), sensitizes cells to UVA as it can catalyze the formation of toxic oxygen-containing radicals (Cabantchik, 2014, Hider and Kong, 2013) such as hydroxyl radical (^•^OH) via Fenton chemistry (Winterbourn, 2008). This can ultimately overwhelm the cellular antioxidant defense mechanisms and lead to cell damage and death (Ma et al., 2015, Reelfs et al., 2004, Reelfs et al., 2010).

Originally, LIP was considered to be cytosolic; however, recent studies have indicated that only a small proportion of the intracellular redox-active pool of labile iron (LI) resides in the cytosol (Aroun et al., 2012, Ma et al., 2015). Indeed, because of constant need for LI in various cellular functions, cytosolic LIP is usually rapidly relocated to distinct subcellular compartments, notably lysosomes and mitochondria, which are the dominant sites for iron redistribution as well as iron sequestration and incorporation into proteins (Fakih et al., 2008, Levi and Rovida, 2009, Reelfs et al., 2010, Sohn et al., 2008). Mitochondria require a continuous provision of LI from the cytosol as they are the main site for heme and iron-sulfur cluster synthesis that are then exported to other cell compartments (Hider and Kong, 2013). Several mechanisms appear to account for mitochondrial iron uptake. In addition, mitoferrins 1 and 2 have been involved in iron transport to mitochondria in a range of organisms (Paradkar et al., 2009, Shaw et al., 2006). As a result of iron influx, the concentration of LI in the mitochondrial matrix exceeds significantly that of cytosol (Petrat et al., 2001, Petrat et al., 2002), which makes these organelles highly susceptible to oxidative damage. Consequently, because mitochondria are the major sites of oxygen consumption and LI is also a strong catalyst of harmful ROS formation, the simultaneous presence of oxygen and iron appears to be detrimental to the organelles (Levi and Rovida, 2009). Excessive mitochondria-mediated ROS production can promote oxidative damage to mitochondrial components, impeding key mitochondrial functions, notably adenosine triphosphate (ATP) synthesis, heme synthesis, and iron-sulfur cluster assembly (Aroun et al., 2012, Murphy, 2009). Oxidative damage to mitochondria will also permeabilize the mitochondrial outer membrane resulting in the leakage to the cytosol of cytochrome c. This will activate the cell’s apoptotic or necrotic pathway, depending on the extent of the oxidizing insult (Pourzand and Tyrrell, 1999, Shidoji et al., 1999). In the event of severe oxidative damage to mitochondrial membranes, ATP depletion will lead to cell death via necrosis because apoptosis involves energy-requiring steps, especially in the formation of the apoptosome complex between apoptosis protease activating factor-1 and cytochrome c (Green and Reed, 1998, Leist et al., 1997, Li et al., 1997, Zou et al., 1999).

Mitochondria are endowed with protective mechanisms against iron-catalyzed ROS-induced damage. Nevertheless, these organelles remain highly sensitive to oxidative damage as deregulation in mitochondrial iron homeostasis has been linked to the development of pathological conditions and cell death (Kakhlon et al., 2010, Levi and Rovida, 2009). In this context, we have shown that UVA radiation or H_2_O_2_ treatment of human skin fibroblasts and Jurkat T leukemia cell line in culture leads to both immediate oxidative damage to mitochondrial membrane and a concomitant rise in the cytosolic LIP (Al-Qenaei et al., 2014, Yiakouvaki et al., 2006, Zhong et al., 2004). As a result of mitochondrial membrane damage, the electron chain reactions are disrupted leading to the excessive generation of ROS and mitochondrial ATP depletion that ultimately causes necrotic cell death. Additionally, it has been shown that both UVA and H_2_O_2_ induce oxidative damage to mitochondrial DNA, with potential implication in photoaging, and skin cancer (Berneburg et al., 1999, Krishnan and Birch-Machin, 2005, Oyewole et al., 2014).

Because mitochondria are a major source of ROS, much effort has been focussed on the design of mitochondria-targeted antioxidant compounds such as MitoQ, a ubiquinone derivative conjugated to triphenylphosphonium that enables this molecule to enter the mitochondria (James et al., 2007). An unrelated compound, 4,5-dihydroxy-1,3-benzenedisulfonic (tiron), a catechol-based metal sequestering agent with ROS scavenging properties, has been described as being able to permeabilize the mitochondrial membrane and thereby be localized in the mitochondrion from the cytoplasm (Krishna et al., 1992, McArdle et al., 2005, Tulah and Birch-Machin, 2013). Such antioxidants have been shown to protect against UVA- and H_2_O_2_-induced mitochondrial DNA damage. Despite the attractive nature of these protective strategies, such “conventional” antioxidants that scavenge mitochondrial ROS will have only a limited protective effect in the skin particularly at the level of mitochondria. This is because they cannot properly address the presence of excess LI in the organelle that is the major contributor to the generation of highly reactive ROS and oxidative damage especially on exposure of cells to strong oxidizing agents such as UVA (Reelfs et al., 2010).

The challenge of countering the effects of the combination of UVA and iron-catalyzed excess ROS formation in the mitochondria highlights a clear need for the development of mitochondria-targeted iron chelating agents to adjust and remove the excess harmful pool of mitochondrial LI. Limited exposure of cultured fibroblasts and keratinocytes to the strong iron chelator desferrioxamine protects skin cells against biologically relevant UVA doses. Nevertheless, despite being a strong iron chelator, desferrioxamine is not an adequate choice for use in skin photoprotection via topical application, due to its hydrophilicity and elevated molecular weight (Reelfs et al., 2010). Although bidentate iron chelators (e.g., catechols and hydroxypyridinones) possess adequate lipophilicity and molecular weight, they have a weak iron scavenging ability at low iron concentrations. This is a major drawback in their use for skin photoprotection via topical application (Liu and Hider, 2002). We therefore decided to opt for hexadentate analogs as these compounds would have similar structures to siderophores (Hider and Zhou, 2005) and would have a high iron scavenging ability despite possessing elevated molecular weight.

A powerful mitochondria-targeted hexadentate tricatechol-based iron chelator was synthesized and evaluated in cells in culture for its protective effects against UVA-induced iron-catalyzed oxidative damage and cell death. We demonstrate that this compound is capable of protecting mitochondria and thereby skin cells from the deleterious effects of the UVA component of sunlight.

## Results

### Compound design and synthesis

We have recently demonstrated the successful use of mitochondria-targeted “SS-peptides” for the delivery of iron-specific sensors to the mitochondria (Abbate et al., 2015a, Abbate et al., 2015b). We therefore chose the same approach to target an iron chelator to the mitochondria.

The catechol-based hexadentate iron chelator described in this study possesses the following properties: (i) high affinity for iron (pFe > 25) and (ii) the iron complexes do not redox cycle at physiological pH. These properties render compound **2** suitable for skin photoprotection.

**Figure 1:**
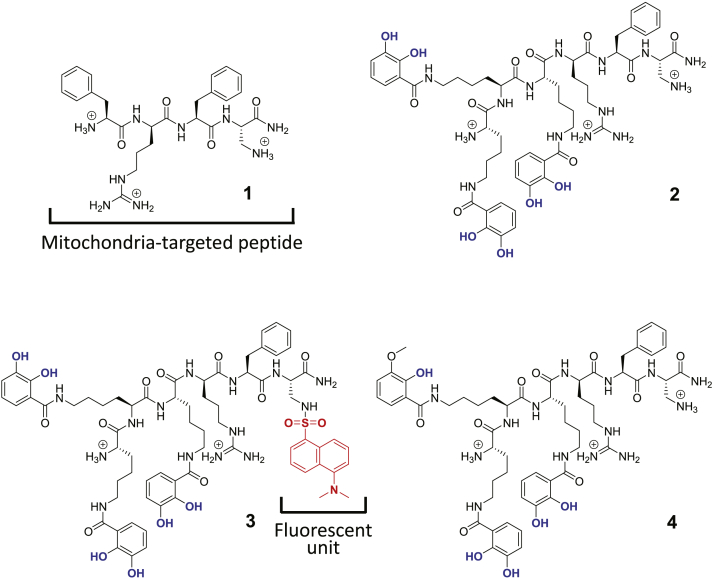
**Structures of compounds 1–4.** The structures of the compounds investigated are depicted with their ionized functions at pH 7.4. Iron-binding functions are indicated in blue. The dansyl (Dns) fluorophore is indicated in red.

Supplementary Table S1 online and Figure 1 document the structures of the compounds investigated in this study. Compound **1** is a simple SS-type mitochondria-targeted peptide that we designed to form the leading mitochondrial address signal to which we have linked a hexadentate chelator to form compound **2**. Compound **3** is a fluorescent version of compound **2** possessing a dansyl fluorophore that renders it possible to follow its subcellular localization by fluorescence microscopy. We have previously shown that the dansyl fluorophore (absorption and emission maxima: 330 and 546 nm, respectively) is a suitable choice as it does not alter the biological function of the chelator peptide (Abbate et al., 2015a, Abbate et al., 2015b). Finally, in compound **4**, hemimethylation of one of the catechol groups renders this compound less efficient at binding iron than its counterpart compound **2**. Supplementary Table S2 online provides some relevant physicochemical properties of the compounds and Supplementary Figure S1 online their HPLC profiles. Although the clogD_7.4_ value for compound **2** is low (−2.05, Supplementary Table S2), it is three orders of magnitude larger than that of the parent peptide **1** (−5.39). Peptide **1** is able to penetrate membranes with relative ease (Zhao et al., 2004) probably by the formation of lipid-peptide complexes. We anticipate that compound **2** will have similar properties especially as it is markedly less hydrophilic than peptide **1**.

### Subcellular localization of the chelator peptide in human primary skin fibroblasts

**Figure 2:**
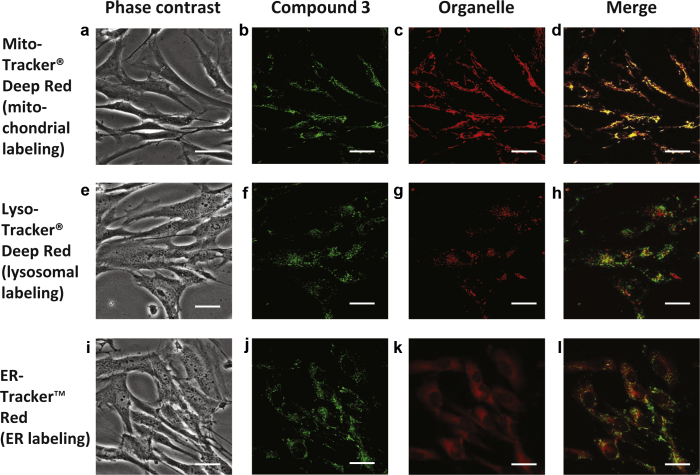
**Microscopy images of subcellular localization studies of the DNS-labeled peptide-chelator H-Hbl-Hbl-Hbl-r-F-DAP(Dns)-NH_2_ (compound 3).** Representative microscopy images are shown, of cells stained with compound **3** in combination with markers for mitochondrial (**a–d**), lysosomal (**e–h**), and ER (**i–l**) compartments. Fluorescence data were collected and analyzed as described in the Materials and Methods section. Green (**b, f, j**) and red (**c, g, k**) fluorescence data were merged together (**d, h, l**) to identify colocalization in yellow. The phase contrast images (**a, e, i**) corresponding to the fluorescence data are also shown. Scale bar = 10 μm. Dns, dansyl; ER, endoplasmic reticulum.

Using fluorescence microscopy, we first investigated the subcellular localization of compound **3** in primary skin fibroblast FEK4 cells. Cells were incubated with compound **3** and costained with a fluorescent marker specific for mitochondria, lysosomes, or endoplasmic reticulum as described in the “Cell biology” section in Supplementary Materials and Methods online. Colocalization of compound **3** to specific organelles was evaluated qualitatively by (i) the occurrence of composite fluorescent yellow signal generated by the cooccurrence of both green and red fluorescent signals above a threshold level (Figure 2) and (ii) intensity profile comparison (Supplementary Figure S2 online). Quantitative evaluation of the extent of colocalization was performed as described in Supplementary Materials and Methods (Supplementary Figure S3 online). The results of both qualitative and quantitative analyses revealed that compound **3** was able to penetrate cells and accumulate preferentially into mitochondria (Figure 2, panels a–d). This tropism for mitochondria was specific, as we could not observe any colocalization with lysosome/endosome (Figure 2, panels e–h) or endoplasmic reticulum compartments (Figure 2, panels i–l). The same subcellular pattern of localization was observed for compound **3** using FCP7 (primary skin fibroblasts from another donor), which we have previously studied in our laboratory (Zhong et al., 2004). Representative pictures and intensity profiles are presented in Supplementary Figure S4 online.

### UVA-induced cell death is prevented by the tricatechol peptide

To highlight the photoprotective role of iron chelation strategy in exposure to solar UV, we chose to use a broad-spectrum UVA lamp (320–400 nm) rather than the whole UV spectrum as a light source. This has the notable advantage to allow us to demonstrate the UVA specificity of the biological effects observed.

To evaluate the photoprotective capability of compound **2** against UVA-induced cell death, we chose the UVA dose of 500 kJ/m^2^, which is equivalent to 140 minutes’ uninterrupted sun exposure at sea level (Pourzand et al., 1999). This is a high but still physiological dose, typically met in recreational sun exposure during a sunny holiday. FEK4 cells were preincubated (or not) with compound **2** and UVA-irradiated.

**Figure 3:**
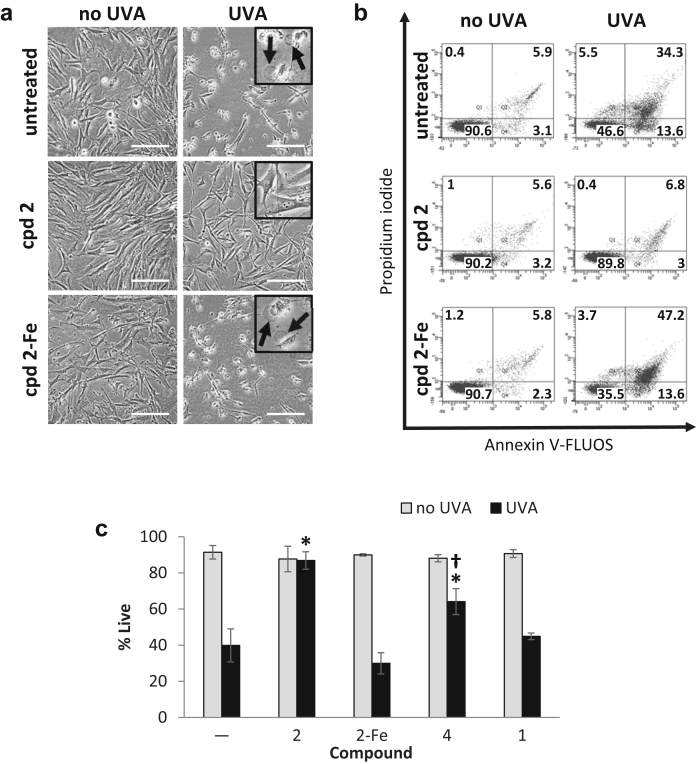
**Compound 2 protects FEK4 cells from UVA-induced cell death.** Cells were treated with compound **2** (cpd **2**) alone or as a complex with iron (cpd **2**-Fe) or UVA or combinations thereof. (**a**) Bright-field images were captured 24 hours after treatment. Swelling (arrows in inset) is indicative of cell death by necrosis and is visible after UVA treatment alone or in combination with cpd **2**-Fe. Scale bar = 50 μm. (**b**) Cells treated as described above were processed for flow cytometry analysis as described in the Materials and Methods section. Dot plots of a representative experiment are shown. Live cells are defined as Annexin V-negative/PI-negative (lower left-hand quadrant). (**c**) Bar chart of flow cytometry results, expressed as means ± SD of percentage live cells from 3 to 5 experiments. *Significantly different (*P* < 0.05) from UVA alone. ^†^Significantly different (*P* < 0.05) from cpd **2**+UVA. UVA, ultraviolet A.

Figure 3a shows phase contrast microscopy images of the morphological changes occurring after various treatments, 24 hours after irradiation. UVA-irradiated cells exhibited significant swelling that is typical of necrosis (Figure 3a). The morphology of cells treated with compound **2** alone (cpd **2**) or followed by UVA irradiation (cpd **2** + UVA) was indistinguishable from that of untreated control cells (no UVA). When cells were pretreated with a complex of compound **2** saturated with iron before irradiation, the damage incurred appeared similar to that observed with UVA alone. Taken together, these results demonstrate that the mitochondria-targeted compound **2** is capable of protecting the cells against UVA-induced damage. This unprecedented level of protection appears to be due to the strong iron-scavenging activity of compound **2**, because it is abrogated on complexation of compound **2** with iron (cpd **2**-Fe).

We also scored the percentages of live, apoptotic, and necrotic cells by flow cytometry using dual staining with Annexin V-FLUOS/propidium iodide 24 hours after UVA treatment (see Supplementary Materials and Methods). Representative Annexin V versus PI scatter plots are shown in Figure 3b and the percentages of live cells are plotted in Figure 3c. Untreated control, cpd **2**-treated and cpd **2**-Fe-treated cells all exhibited similar high levels of live populations (ca 90%, Figure 3b). UVA induced predominantly necrotic cell death (ca 40%, Figure 3b), consistent with the morphologic changes observed microscopically. Furthermore, the percentage of cells undergoing apoptosis in UVA-irradiated samples remained low (ca 14%, Figure 3b). Compound **2** (50 μM) afforded full protection against UVA-induced cell death as seen by restoration of percentage of live cells to control levels (Figures 3b and c). Photoprotection was concentration-dependent and could be observed with as low as 5 μM compound **2** (Supplementary Figure S5 online). Compound **4**, a form of compound **2** less able to bind iron, afforded half of the protection provided by the latter, while compound **1** (with no iron-binding activity) had no effect on cell survival when incubated with cells before UVA irradiation (Figure 3c). Finally, in line with the morphological observations made in Figure 3a, pretreatment of cells with the “inactive” (iron-complexed) chelator peptide failed to protect them against UVA-induced necrotic cell death (Figures 3b and c). The complex per se did not generate any significant toxicity. The protective effects observed with compound **2** were reproducible with the dansyl-labeled version compound **3**, indicating that the dansyl fluorophore does not alter the biological function of the chelator peptide, as previously observed (Abbate et al., 2015a). In FCP7 fibroblasts, compound **2** afforded full protection against UVA radiation in an iron-dependent fashion (Supplementary Figure S6a online), confirming the results obtained with FEK4 cells.

### The tricatechol peptide prevents the loss of mitochondria membrane potential associated with UVA irradiation

Loss of mitochondrial membrane potential is an early consequence of UVA radiation-mediated oxidative damage to mitochondrial membrane (Pourzand and Tyrrell, 1999, Zhong et al., 2004). Because compound **2** localizes specifically to the mitochondria, we sought to identify if its mechanism of protection involved the prevention of mitochondrial membrane depolarization. For this purpose, FEK4 cells were preincubated (or not) with compound **2** and irradiated with 500 kJ/m^2^ UVA. They were then incubated with the mitochondria-specific red fluorescent cationic dye tetramethylrhodamine methyl ester (TMRM) and analyzed by flow cytometry 2 and 24 hours after UVA. A decrease in TMRM fluorescence is indicative of mitochondrial membrane depolarization.

**Figure 4:**
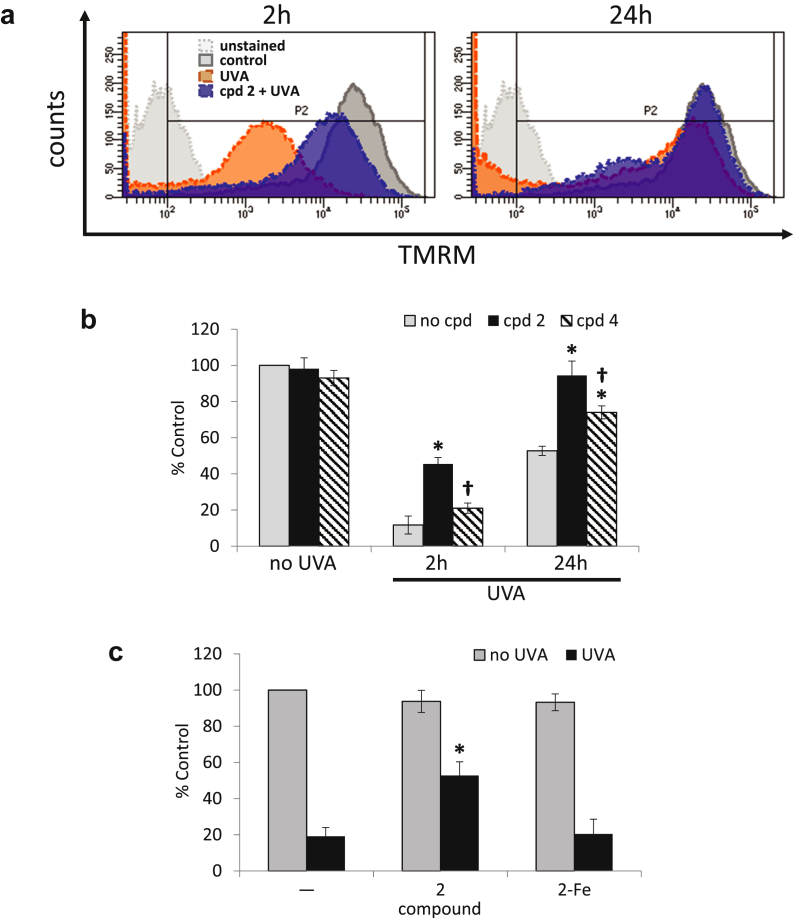
**Compound 2 significantly reduces UVA-induced damage to mitochondria membrane.** (**a**) Representative fluorescence intensity profiles of samples stained with TMRM at 2 and 24 hours after UVA irradiation as described in the Materials and Methods section. (**b**) Bar chart of the results of TMRM staining experiments. Fluorescence median intensities (MFIs) collected over the region delineated in panel **a** as P2 are expressed as percentage of untreated control. *Significantly different (*P* < 0.05) from the corresponding UVA-treated sample. ^†^Significantly different (*P* < 0.05) from the corresponding cpd **2**+UVA-treated sample. (**c**) FEK4 cells were pretreated with either compound **2** alone or as a complex with iron, before UVA irradiation and ATP production was measured as described in the Materials and Methods section. Results are expressed as percentage of untreated control. *Significantly different (*P* < 0.05) from the UVA alone sample. ATP, adenosine triphosphate; TMRM, tetramethylrhodamine methyl ester; UVA, ultraviolet A.

The results (Figures 4a and b) revealed that UVA induced a dramatic drop in TMRM fluorescence already at the 2-hour time point. This drop was similar to the depolarization caused by the mitochondrial membrane uncoupling compound carbonyl cyanide 4-(trifluoromethoxy)phenylhydrazone (FCCP) (data not shown). However, pretreatment with compound **2** provided a significant protection at that time point. While at 24-hour time point, there was a partial but noticeable recovery of TMRM fluorescence in cells treated with UVA, full recovery of membrane potential could be observed in compound **2**-treated cells. In contrast, compound **4** was not able to prevent significantly the UVA-induced decrease in mitochondrial membrane potential at 2 hours after irradiation, in line with its lower iron-binding capability. Nevertheless, it afforded an intermediate level of protection at the 24-hour time point, although less marked than that observed with compound **2**. Taken together, these results show a direct link between the UVA-induced mitochondrial membrane damage and necrotic cell death, both of which could be fully prevented by pretreatment with the mitochondrial iron chelator compound **2**. Similar results were obtained using FCP7 cells (Supplementary Figure S6b), albeit revealing a higher sensitivity to UVA-induced mitochondrial damage when compared with FEK4 cells.

### The protective effect of mitochondria-targeted chelator compound **2** on ATP depletion associated with UVA-mediated necrosis

We investigated if the protection of the mitochondrial membrane potential afforded by compound **2** on UVA irradiation correlated with a protection against ATP depletion. The measurement of the intracellular level of ATP 4 (data not shown) and 24 hours after UVA irradiation (Figure 4c) showed that UVA irradiation depleted up to 80% of ATP levels compared with unirradiated control cells, as previously observed (Zhong et al., 2004). Pretreatment of cells with compound **2** significantly recovered the ATP levels to 60% of the control level at both time points (Figure 4c and data not shown). This level of protection against ATP depletion appears to be sufficient to prevent necrotic cell death induced by UVA (Figure 3). Furthermore, the protection afforded by compound **2** against UVA-induced ATP depletion appeared to be iron-dependent because its complexation with iron abolished its protective effect. In a similar fashion to FEK4 cells, compound **2** diminished UVA-induced ATP depletion (Supplementary Figure S6c) in FCP7 cells.

## Discussion

We have shown that the mitochondria-targeted compound **2** provides an unprecedented protection against oxidative damage and cell death induced by a high but environmentally relevant dose of UVA in primary skin fibroblast cells. The abrogation of cell death by these chelator peptides is entirely iron-dependent, because their iron saturation abolished their photoprotective effect. Furthermore, partial impairment of the iron binding capability of one of the catechol moieties in compound **4** significantly reduced its photoprotective ability (Figure 3c).

We chose an “SS-like peptide” for the effective delivery of our hexadentate iron chelator to the organelle, due to the known mitochondrial selectivity of this family of peptides (Abbate et al., 2015a, Abbate et al., 2015b, Szeto, 2008, Szeto and Schiller, 2011, Zhao et al., 2003). These peptides as well as some recently described cationic and lipophilic peptides (Horton et al., 2008, Yousif et al., 2009) have been used successfully as carriers of functional molecules (including antioxidants or drugs) to the mitochondria (Pereira and Kelley, 2011, Zhao et al., 2004, Zhao et al., 2005). These aromatic-cationic peptides, unlike MitoQ, are not taken up due to mitochondrial membrane potential (Δψ_m_) as they are reported to be concentrated in depolarized mitochondria (Cerrato et al., 2015, Doughan and Dikalov, 2007).

It is essential that the iron chelating peptides we describe herein do not interfere with the normal cellular ferrokinetic pathways; otherwise serious toxic side effects could be anticipated. Hexadentate catechols are highly selective for iron(III), the oxidation state of iron that accumulates in situations of both iron overload and the uncontrolled lysis of lysosomes. Whereas the cytosol contains a LIP of iron(II), the lysosomes possess a high content of ferritin, which in turn contains iron(III). Although hexadentate catechols can autoxidize iron(II), the rates of this reaction are extremely slow at iron concentrations of 1–2 μM, which are typically found in mammalian cell cytosol (Hider and Kong, 2013). Thus, we do not anticipate appreciable interference with the cytosolic iron pool or mitochondrial iron pool, which is also dominated by iron(II), under normal physiological conditions (Ma et al., 2015). A similar situation occurs with deferiprone, a hydroxypyridin-4-one, which has been used for over 20 years in clinical practice for the treatment of iron overload. Like hexadentate catechols, deferiprone is highly selective for iron(III). Although it facilitates the autoxidation of iron(II), it does so at an extremely low rate at low μM concentrations (Devanur et al., 2008).

The role of LI in UVA-induced oxidative damage and necrotic cell death has long been established (Dissemond et al., 2003, Kvam et al., 2000, Morlière et al., 1991, Pourzand and Tyrrell, 1999, Zhong et al., 2004). Earlier studies focused more on oxidant-mediated destabilization of lysosomes (Basu-Modak et al., 2006, Fakih et al., 2008, Pourzand et al., 1999, Yu et al., 2003). However, subsequent studies recognized mitochondria as the principal destination of LI in cells and therefore a primary site of prooxidant generation rendering these organelles particularly susceptible to oxidative damage. Indeed, high levels of chelatable LI have been detected by several groups in the mitochondria of cultured cells using various probes (Glickstein et al., 2005, Petrat et al., 2002, Rauen et al., 2007). Additional studies by us have established that the oxidant-induced destabilization of mitochondrial membrane resulting from intramitochondrial Fenton reactions is one of the early cellular events occurring after UVA irradiation of skin cells (Yiakouvaki et al., 2006, Zhong et al., 2004). This provokes a loss of mitochondrial membrane potential and ATP depletion leading irreversibly to necrotic cell death (Green and Reed, 1998, Zou et al., 1999) that can be prevented by iron chelators such as salicylaldehyde isonicotinoyl hydrazone. This is due to the capability of these compounds to not only deplete the cytosolic LIP but also access and sequester the loosely available LI in both lysosomal and mitochondrial compartments (Pelle et al., 2011, Simunek et al., 2005, Yiakouvaki et al., 2006). An inevitable consequence of prolonged nontargeted chelating activity of such chelators is unwanted cytotoxicity (Reelfs et al., 2010, Yu et al., 2006). Compound **2**, which is entirely localized to the mitochondria, is expected not to display such undesired side effects. Indeed, time-course studies performed in our laboratory comparing compound **2** and salicylaldehyde isonicotinoyl hydrazone show that unlike salicylaldehyde isonicotinoyl hydrazone, compound **2** does not exhibit any cytotoxicity at the highest tested concentration of 100 μM up to 48 hours’ exposure (Supplementary Figure S7 online). Our results in the two cell lines FEK4 and FCP7 clearly show that compound **2** is fully protective against UVA-induced necrotic cell death despite showing a partial protection against ATP depletion. This phenomenon has already been observed in an unrelated study with Jurkat T cells treated with H_2_O_2_ showing that iron chelators were effective at protecting cells against necrotic cell death yet not being fully effective in protecting against ATP depletion. This suggests that oxidant-induced ATP depletion is only partially iron-dependent. A plausible iron-independent mechanism would involve oxidant-induced damage to mitochondrial respiratory chain proteins. Because both UVA and H_2_O_2_ are glutathione-depleting agents (Al-Qenaei et al., 2014, Lautier et al., 1992), protein oxidative damage would be exacerbated under these conditions. Further studies are necessary to unravel these iron-independent mechanisms, but this is out of the scope of this study. Nevertheless, it appears that after UVA or H_2_O_2_ treatment, cells may only need to reach a threshold level of ATP production to be able to fully evade necrotic cell death. This is strengthened by the observation that partial replenishment of ATP by glucose supplementation can rescue skin fibroblasts from UVA-induced necrotic cell death (Zhong et al., 2004).

The importance of mitochondrial LI in oxidative injuries has also been demonstrated in a series of genetic disorders with defective cellular iron utilization (i.e., trafficking and incorporation into proteins) resulting in a toxic increase in iron concentrations in mitochondria of excitable cells often leaving the cytosol iron-depleted (Kakhlon et al., 2010, Richardson et al., 2010). For example, cultured fibroblasts from Friedreich’s ataxia patients that contain a high mitochondrial iron level have been shown to be highly sensitive to iron stress and significantly more sensitive to H_2_O_2_-induced cell death than fibroblasts from healthy individuals (Lim et al., 2008, Wong et al., 1999). The highly specific and mitochondria-targeted iron chelator designed in this study should therefore be beneficial for the therapy of mitochondrial iron-related oxidative injuries and pathological conditions such as Friedreich’s ataxia. Although recently a series of mitochondria-targeted antioxidants have been proposed for the therapy of mitochondria-related pathologies (Jauslin et al., 2003, Jauslin et al., 2007, Zhao et al., 2004, Zhao et al., 2005), to our knowledge a highly specific mitochondria-targeted iron chelator such as the one introduced here has not yet been reported.

A variety of iron chelator molecules have been patented as part of sunscreen formulations (Reelfs et al., 2010). Although their mild iron chelating properties provide protection against UVB-induced damage, studies from this laboratory and others have demonstrated that these compounds are not effective against UVA-induced iron damage (Aroun et al., 2012, Creighton-Gutteridge and Tyrrell, 2002, Reelfs et al., 2010). For efficient protection against UVA-induced iron damage of skin, strong chelators are required. However, these are incompatible with prolonged systemic administration due to toxic effects caused by iron starvation of healthy cells. The mitochondria-targeted chelator-peptide compound **2** provides a solution to this problem and thus can address the current unmet need in the skin care/sunscreen field for potent sunscreen ingredients that are targeted specifically to the principal site of the damage (i.e., mitochondria) in the skin cells and are highly effective UVA photoprotectant. It can be argued that compound **2** may have an effect on iron-sulfur cluster and/or heme synthesis. Although these important issues are out of the scope of this article, we plan to address them in the near future.

In daily life, skin cells are clearly not exposed exclusively to the UVA waveband but to the entire solar spectrum that also includes UVB. We introduce the strategy of iron chelation as photoprotectant with the intent to incorporate the chelating molecule as an ingredient to a sunscreen formulation designed to protect against both UVA and UVB components of sunlight. Current filters provide “passive” protection by absorbing and reflecting harmful UV rays from the skin. Manufacturers have adopted an alternative strategy of adding antioxidants such as vitamins C and E, to their sunscreen products which offers “active” protection by boosting the body’s natural antioxidant reserve to quench any ROS generated from UVA that has passed the UV filters. Despite the attractive nature of this photoprotection strategy, numerous studies in this field have demonstrated that “conventional” antioxidants that scavenge ROS (e.g., vitamins C and E) have only a very modest protective effect (Reelfs et al., 2010). However, studies from our laboratory and others have demonstrated that iron, and in particular mitochondrial iron, plays a major role in the oxidative damage caused by UVA (Aroun et al., 2012). Within this context, we propose that our mitochondria-targeted iron chelator be added as an essential UVA photoprotectant to sunscreen formulations.

## Materials and Methods

### Chelator and peptide synthesis

See Supplementary Materials and Methods for protocols and characterization information.

### Reagents

For cell cultivation, all reagents used were cell culture-grade and purchased from Life Technologies (Paisley, Scotland), except foetal calf serum, which was from Life Technologies (Paisley, UK) and phosphate buffered saline from Oxoid (Basingstoke, UK). TMRM and the organelle-specific markers MitoTracker Deep Red FM, Lyso Tracker Deep Red, and endoplasmic reticulum Tracker Red were purchased from Invitrogen (Paisley, Scotland). Annexin V-FLUOS was from Roche (Welwyn Garden City, UK). All other reagents were from Sigma-Aldrich (Gillingham, UK).

### Cell culture

The human primary fibroblasts FEK4 and FCP7 were a kind gift from Prof. Tyrrell’s laboratory and were grown as described previously (Abbate et al., 2015a). The foreskin fibroblast cultures were initiated in Prof. Tyrrell’s laboratory, Switzerland, from tissue samples obtained in full compliance with ethical regulations and legislation in force at the time and respecting complete donor confidentiality. Patient consent for experiments was not required because under Swiss laws in force at the time, human material left over from surgery was considered as discarded material. These cells have been isolated from foreskin biopsies at passage 0. From that, subcultured and frozen stocks between passage 1 and 12 were available for the present study.

### Treatments and peptide delivery

The (chelator) peptides were prepared as 100 mM stock solutions in DMSO. They were diluted to the desired final concentration (50 μM unless specified) and added to cells overnight as described previously (Abbate et al., 2015a, Abbate et al., 2015b).

### UVA irradiation

Irradiations were performed in phosphate buffered saline at approximately 25 °C using a broad spectrum Sellas 4 kW UVA lamp (Sellas, Germany), as described previously (Pourzand et al., 1999).

### Flow cytometry and live cell microscopy

All flow cytometry- and live cell microscopy-based protocols are described in Supplementary Materials and Methods.

### ATP measurement

ATP production by cells after various treatments was monitored with a luminometer (Turner Designs Model TD-20/20) 24 hours after UVA, using the ViaLight plus kit (Lonza Biologics plc, Slough, UK), as described previously (Al-Qenaei et al., 2014).

### Iron complexation

Iron:ligand complexes were prepared by mixing equimolar amounts of iron(III) as FeCl_3_•6H_2_O and the hexadentate-peptide molecule. The complex was allowed to form for 1 hour at room temperature before incubation with the cells overnight at 37 °C at a final concentration of 50 μM.

### Statistical methods

Results are expressed as the mean ± SD. Significant differences (*P* < 0.05) were determined by either a paired or unpaired *t*-test after one-way analysis of variance.

## Conflict of Interest

The authors state no conflict of interest.
