# Supplementary material for: A Powerful Mitochondria-Targeted Iron Chelator Affords High Photoprotection against Solar Ultraviolet A Radiation
Source: J Invest Dermatol. 2016 Aug;136(8):1692–700. doi: 10.1016/j.jid.2016.03.041 (PMC4946793; doi:10.1016/j.jid.2016.03.041)
Supplement: Supplementary Data [file mmc1.pdf]

Supporting Information (ESI) for

A powerful mitochondria-targeted iron chelator affords high photoprotection against solar ultraviolet A radiation

Olivier Reelfs[1]*, Vincenzo Abbate[2]*, Robert C. Hider[2] and Charareh Pourzand[1]**

[1] Department of Pharmacy and Pharmacology, University of Bath, Claverton Down, Bath BA2 7AY, United Kingdom.
[2] Institute of Pharmaceutical Science, King's College London, Franklin-Wilkins Building, 150 Stamford Street, London SE1 9NH, United Kingdom.

* These authors contributed equally.

**Corresponding author: Charareh Pourzand, Department of Pharmacy and Pharmacology, University of Bath, Claverton Down, Bath BA2 7AY, United Kingdom. Email: prscap@bath.ac.uk

SUPPLEMENTARY MATERIALS AND METHODS

General

Ultra-high purity water obtained from a Milli-Q system was used throughout this study for both the chemistry and biology work. NovaPEG Rink Amide resin (substitution = 0.49 mmol/g) was supplied from Merck Chemicals Ltd (Nottingham, UK). Solvents were from Fisher Scientific UK Ltd (Loughborough, UK). Fmoc-protected amino acids and all other peptide synthesis reagents were from Bachem (Merseyside, UK). All the other chemicals were supplied from Sigma-Aldrich (Gillingham, UK). All reagents were used as received except DMF which was kept under a constant flow of nitrogen. The peptides described in this work were prepared in a similar manner to that recently described.

Analytical

The analytical RP-HPLC was carried out on a HP1050 HPLC system equipped with an autosampler, a quaternary pump and a Diode-Array Detector. A Zorbax SB C-18 2.1 mm x 10 cm (particle size 3.5 micron) column was employed. The flow rate was 0.2 mL/min and the eluents were monitored at wavelengths between 210-280 nm. A linear gradient of mobile phase B (acetonitrile containing 0.1% TFA) over mobile phase A (0.1% TFA in water) from 0-90% B in 20 minutes was performed. Data were collected and analyzed using ChemStation software.

The semi-prep HPLC purification was performed using a X-terra Prep MS C18 Column (5 micron, 10 x 100 mm) operating a flow rate of 7 mL/min. Isolated fractions were reanalyzed via analytical RP-HPLC as above and identical fractions were pooled and lyophilized.

The ESI-MS analyses were performed using a Waters Micromass ZQ mass spectrometer (Manchester, UK).

UV-vis and fluorescence measurements were performed on a Perkin Elmer spectrophotometer and a Perkin Elmer spectrofluorometer, respectively. Peptide solutions were prepared at the appropriate concentrations in 0.1 M MOPS buffer, pH 7.4.

Cell biology

Flow cytometry

Data were obtained using a Becton Dickinson FACSAria III fitted with laser lines of excitation at 405, 488, 561 and 633 nm. Cells were gated via forward scatter versus side scatter dot-plot so as to exclude very small debris in the origin. Data were collected from a minimum of 10'000 cells (events) and analyzed with FACSDiva Software version 8.0.1 (Becton Dickinson, San Jose, CA).

Annexin V /PI labeling: Cell status following the different treatments was examined using the dual staining with Annexin V/PI as described previously (Zhong *et al*., 2004). Populations were determined as percentages via quadrant analysis of dot-plots of Annexin V-FLUOS versus PI. “Live” cells were defined as Annexin V-negative/PI-negative. Double-positive and Annexin V-negative/PI-positive cells were considered “necrotic” and Annexin V-positive/ PI-negative cells as “apoptotic’’.
TMRM labelling: For the measurement of mitochondrial transmembrane potential ($\Delta\psi_m$), at the times indicated post-UVA irradiation, the conditioned medium was removed, cells rinsed with PBS and incubated for 30 min at 37 °C with 50 nM TMRM in phenol-red free Hank's Balanced Salt Solution (HBSS) supplemented with 10 mM 4-(2-hydroxyethyl)-1-piperazineethanesulfonic acid (HEPES). The cells were then collected by trypsinization, resuspended in HBSS buffer and median fluorescence intensities (MFI) scored.

Live cell microscopy
Bright-field images used to document morphological changes following treatments were captured on a Motic inverted microscope (model AE2000) (Motic Deutschland GmbH, Wetzlar, Germany) via a Moticam 580 digital camera and a Plan 10x objective. Subcellular localization of the DNS-labelled peptide (compound **3**) was investigated on an Olympus IX51 inverted epifluorescence microscope equipped with a 100 W mercury UV lamp. Cells were imaged with a 40x oil objective (UAPONO340-2). Images were acquired via an Olympus DP72 digital camera controlled by Olympus cell^P Analysis Image Processing software (Soft Imaging System GmbH, Muenster, Germany). Cells showing yellow staining, resulting from superimposition of the chelator peptide-specific green signal and the organelle-specific red signal, were qualitatively considered positive for colocalization. The co-occurence in the images, of green and red fluorescent signals above a threshold level was further confirmed by analysis of intensity profiles collected across cells using cell^P (from Olympus) Image Processing software. The extent of colocalization of the fluorescent compound **3** with mitochondria, lysosomes or ER compartments was also assessed quantitatively by Manders' correlation coefficients (MCCs) M1 and M2 using ImageJ software with the JaCoP plug-in (Bolte and Cordelières, 2006; Manders *et al*., 1993) from a random selection of image fields as described previously (Abbate *et al*., 2015a). MCCs provide a measure of how much of the signal intensity of a channel occurs in the same location as the other channel. Thus M1 represents the extent of overlap of compound signal (green) with the organelle signal (red), whereas M2 represents the amount of organelle signal overlapping the compound signal. MCCs values range from zero (uncorrelated distributions of two probes with one another) to one (fully correlated distributions of two probes). MCCs were chosen instead of the Pearson's colocalization coefficient as they are particularly well suited when the fluorescent signals distribute to different types of compartments (Dunn *et al*., 2011).

MTT assay
Cells grown in 96 well plates were treated as indicated in the figure legend. At the end of the treatment, the media was removed and the cells were incubated for 3 h at 37 °C with 100 μL serum-free media containing MTT (3-[4,5-dimethylthiazol-2-yl]-2,5-diphenyl-tetrazolium bromide) at a concentration of 0.5 mg/mL. The MTT solution was then removed and 50 μl DMSO was added to the cells in order to dissolve the formazan formed. Absorbance was read at 550 nm using a spectrophotometer VERSAmax^{TM} (Molecular devices, California). The percentage of viable cells was calculated by comparing the absorbance of treated *versus* non-treated control cells.

SUPPLEMENTARY TABLE AND FIGURES

Table S1: Peptides described in manuscript.

Compound	Sequence
**1**	H-F-r-F-DAP-NH_2
**2**	H-Hbl-Hbl-Hbl-r-F-DAP-NH_2
**3**	H-Hbl-Hbl-Hbl-r-F-DAP(Dns)-NH_2
**4**	H-Hbl(OMe)-Hbl-Hbl-r-F-DAP-NH_2 [1]

The four peptides possess both L and D aminoacids and are C-amidate in order to prolong biological lifetimes. Compounds **2**, **3** and **4** all contain iron(III) coordinating centres.

Hbl: modified amino acid Nε-2.3-dihydroxybenzoyllysine; Dns: dansyl fluorophore; F = Phe; r = D-Arg; DAP = diaminopropionic acid.

[1] This product contains a mixture of isomers, all possessing a single methoxy unit across the six phenolic moieties, as judged by mass spectrometry data.

Table S2: Physico-chemical properties of the peptides studied.

Compound	Sequence	MW	Found m/z	cLogD$_{7.4}$ [2]
**1**	H-F-r-F-DAP-NH_2	553.7	554.3	-5.39
**2**	H-Hbl-Hbl-Hbl-r-F-DAP-NH_2	1199.3	1199.5	-2.05
**3**	H-Hbl-Hbl-Hbl-r-F-DAP(Dns)-NH_2	1432.6	1432.4	1.23
**4**	H-Hbl(OMe)-Hbl-Hbl-r-F-DAP-NH_2 [1]	1213.4	1213.0	-1.97

Compound **1** is a tetrapeptide whereas compounds compounds **2**, **3** and **4** are hexapeptides. The introduction of catechol functions in the peptides markedly decreases their hydrophilicity (increased clogD$_{7.4}$). Indeed, the fluorescent peptide **3** possesses a positive clogD$_{7.4}$ value.
[1] This product contains a mixture of isomers, all possessing a single methoxy unit across the six phenolic moieties, as judged by mass spectrometry data.
[2] Calculated with MarvinView 6.1.3.

Figure S1: HPLC traces of compounds 1-4.

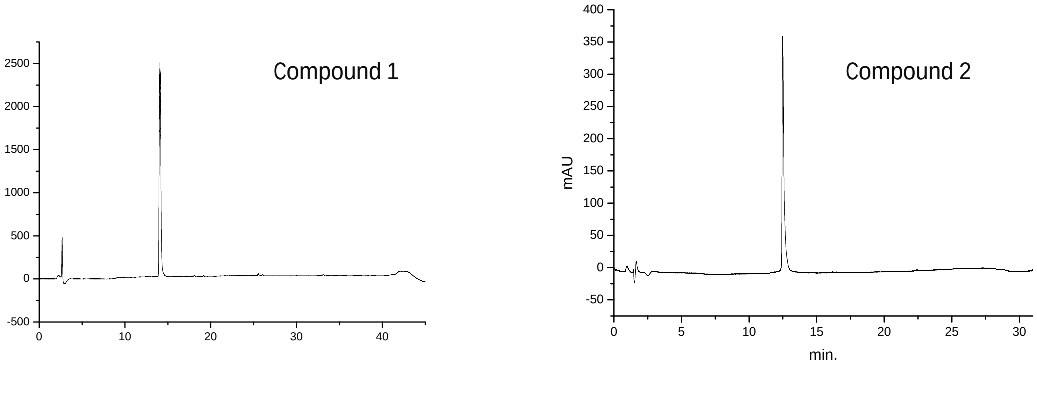

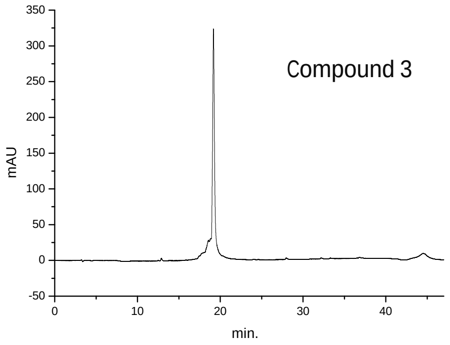

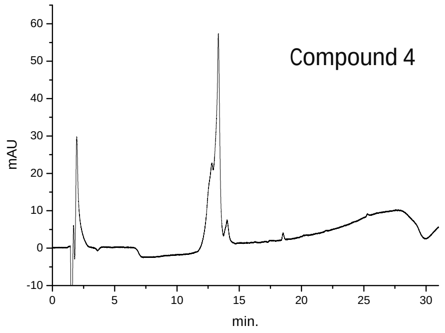

In this study, a reverse-phase HPLC analysis was performed using a C18 column and a gradient of acetonitrile over water to perform the chromatographic separation.

Figure S2: Intensity profiles of microscopy images in FEK4 cells.

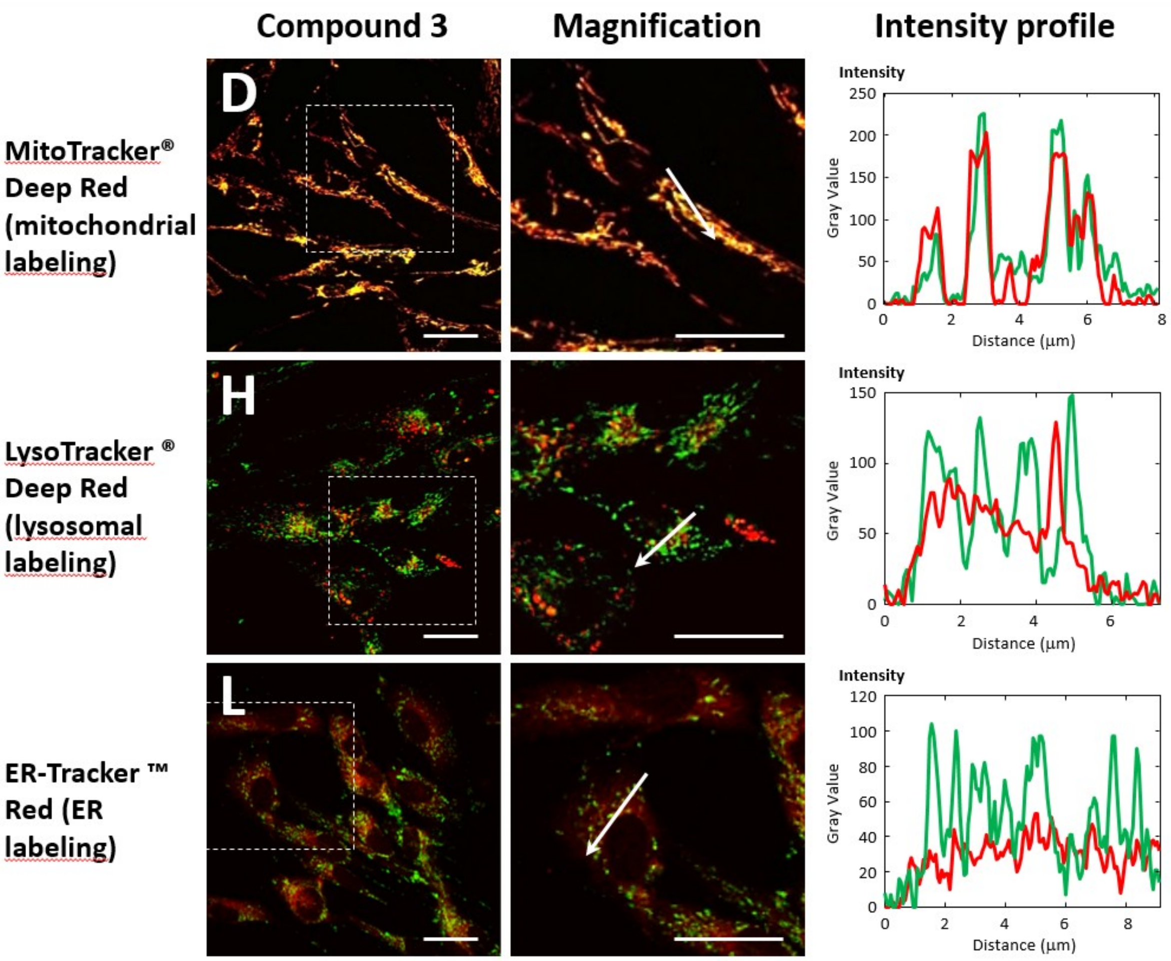

Panels D, H and L from Figure 2 representing the merged channels are shown. Magnifications (x2) correspond to the indicated dotted squares. Representative pixel intensity profiles were collected across field sections indicated by the white arrows. Green profiles depict fluorescent signals from compound **3**, whereas red profiles account for fluorescence from markers for mitochondria, lysosomes or ER compartments. Note the marked overlap between the profiles of compound **3** and mitochondria, contrasting with the lack of correlation between profiles of compound **3** and lysosomes or ER. Scale bar = 10 µm.

Figure S3: Quantitative analysis of colocalization of compound 3 with subcellular organelles in FEK4 cells.

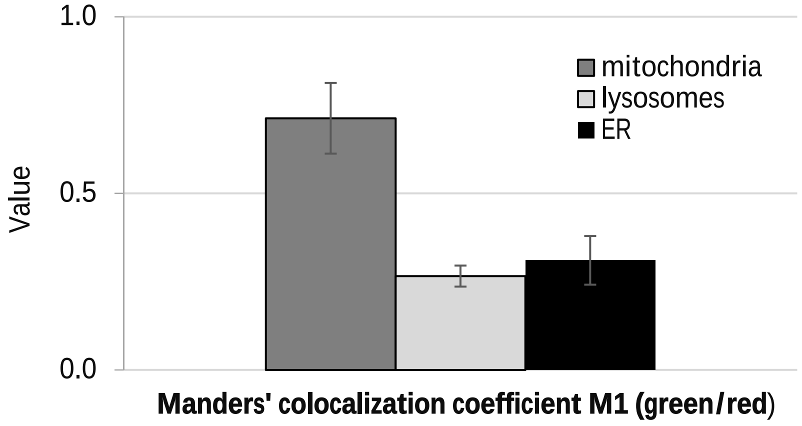

The extent of colocalization of compound **3** with mitochondria, lysosomes or endoplasmic reticulum (ER) compartments was measured quantitatively using Manders' correlation coefficients (MCCs) M1 and M2 using ImageJ software with the JaCoP plug-in as described in Materials and Methods. M1 represents the fraction of signal from compound **3** colocalizing with the signal from the organelles. The values obtained for M1 are presented as means +/- SD per field, calculated from at least two random fields of 8-10 cells over three independent experiments.

Figure S4: Mitochondrial localization of compound 2 in FCP7 cells.

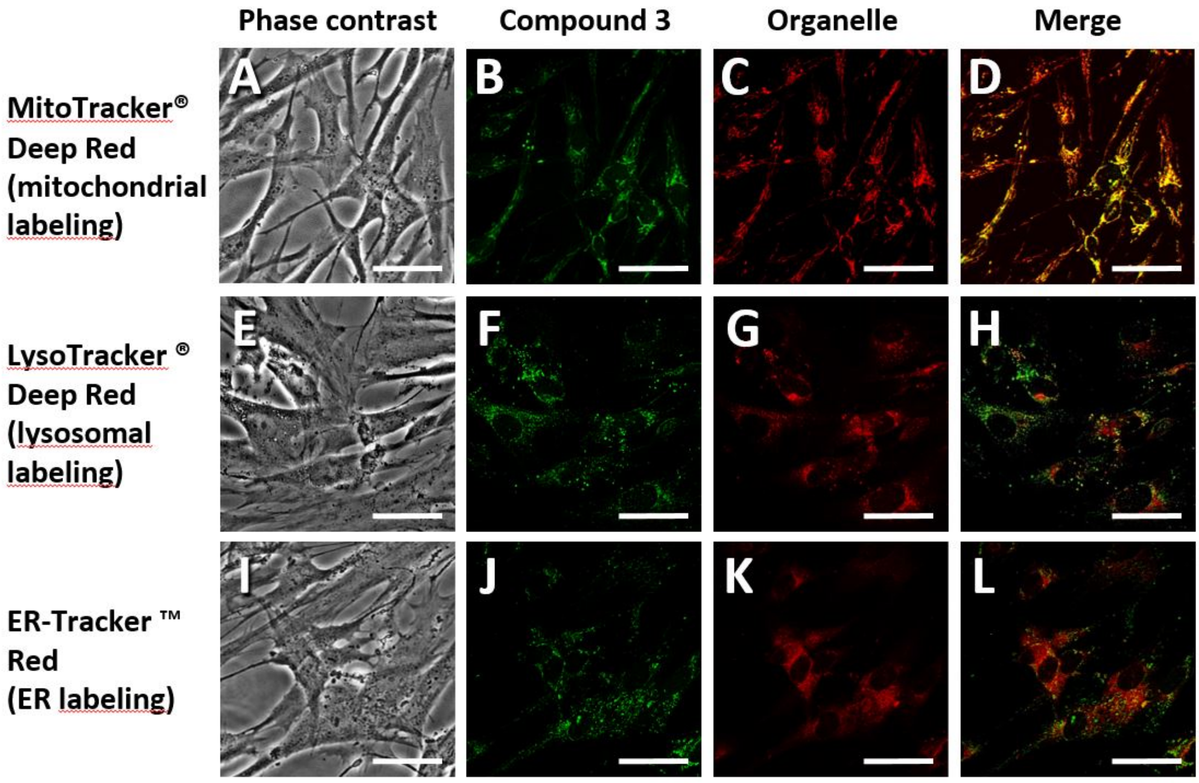

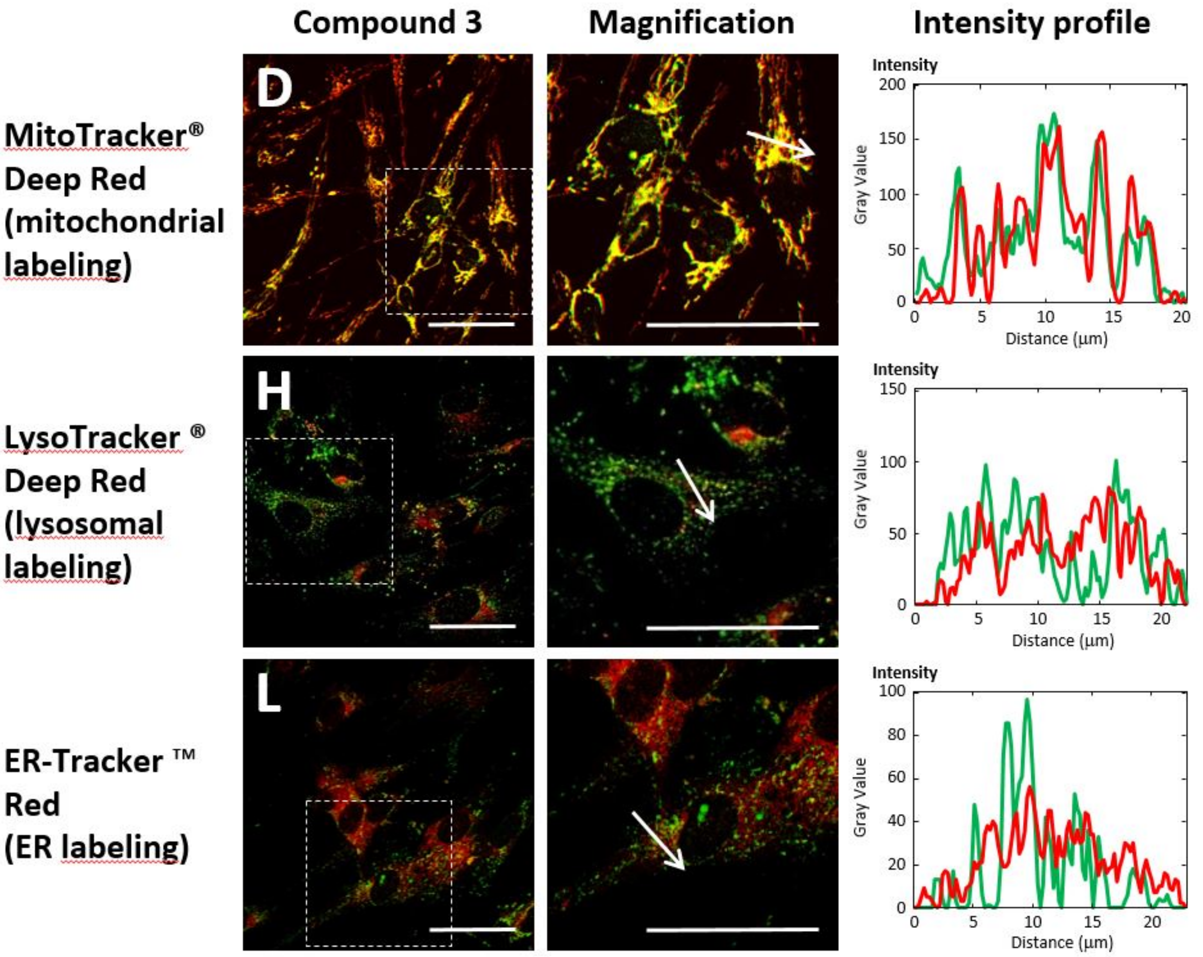

Microscopy images of subcellular localization studies of compound 3 in FCP7 cells. Representative microscopy images are shown, of FCP7 cells stained with compound **3** (fluorescent derivative of compound **2**) in combination with markers for mitochondrial (**A–D**), lysosomal (**E-H**) and ER (**I-L**) compartments. Fluorescence data were collected and analysed as described in Materials and Methods. Green (**B, F, J**) and red (**C, G, K**) fluorescence data were merged together (**D, H, L**) to identify co-localization in yellow. The phase contrast images (**A, E, I**) corresponding to the fluorescence data are also shown. Representative pixel intensity profiles were collected across field sections indicated by the white arrows. Green profiles depict fluorescent signals from compound **3**, whereas red profiles account for fluorescence from markers for mitochondria, lysosomes or ER compartments. Scale bar = 50 µm.

Figure S5: Concentration-dependent photoprotection of FEK4 cells by compound 2.

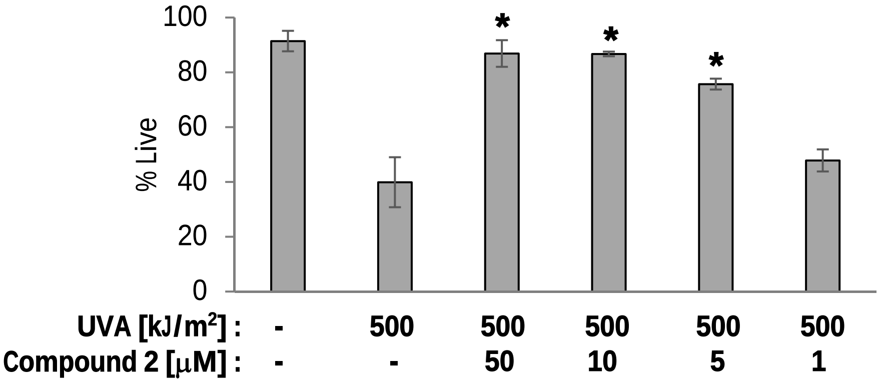

FEK4 cells were treated as indicated and the percentage of live cells (double negative for Annexin V and PI) was determined by flow cytometry using double staining with Annexin V/PI as described in Materials and Methods. Data are represented as means +/- SD from at least three independent experiments. * Significantly different from UVA-irradiated alone (p<0.05).

Figure S6: Photoprotective effects of compound 2 in FCP7 cells.

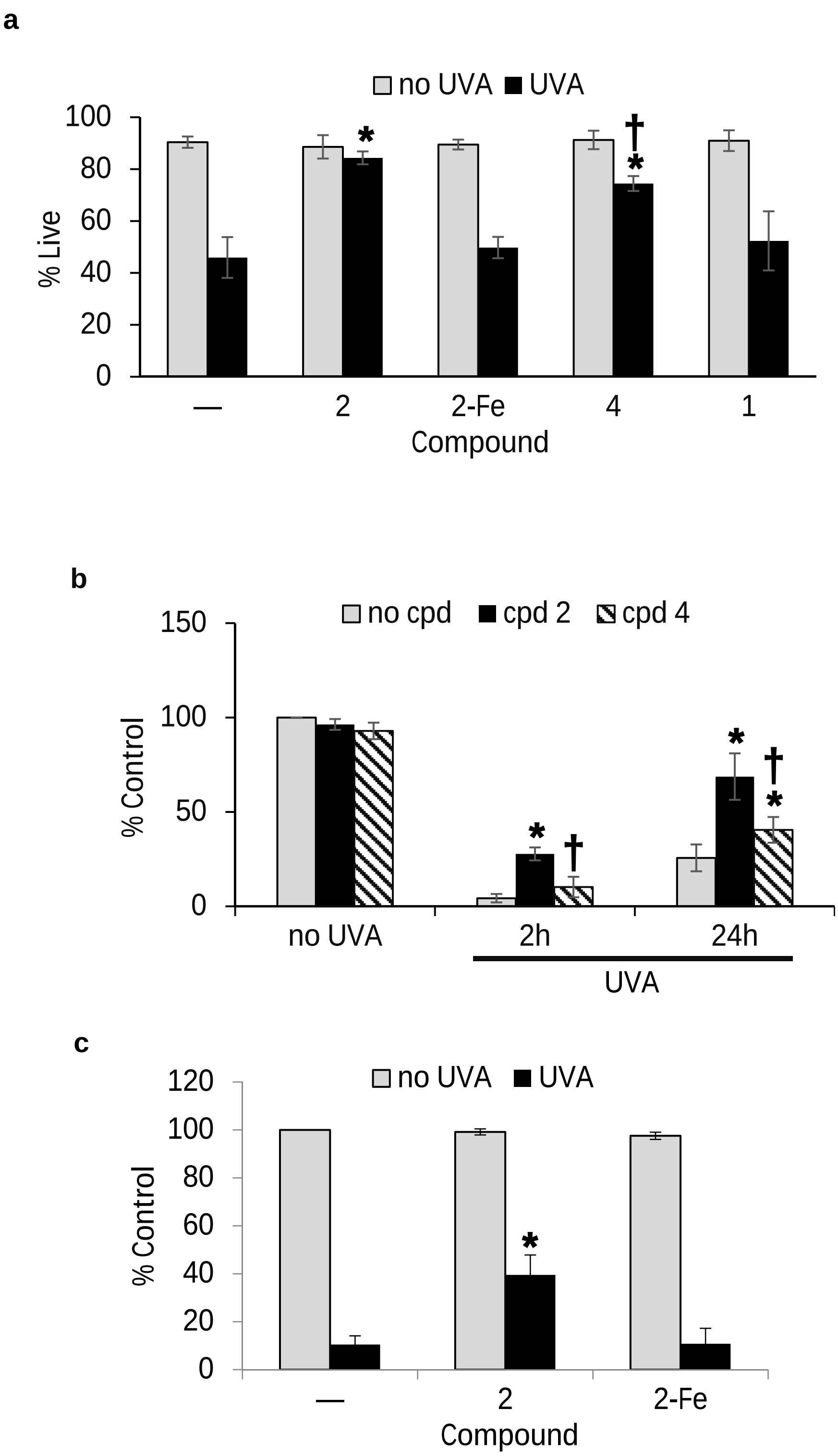

FCP7 cells were treated as indicated in each figure above (see Materials and Methods for details). They were then processed for **(a)** flow cytometry measurement of percentage live

cells by Annexin V/PI staining, **(b)** loss of mitochondrial membrane potential by TMRM fluorescence or **(c)** for ATP measurement, as described in Materials and Methods. Results are represented as bar charts, expressed as means +/- SD from 3-5 experiments. * Significantly different (p<0.05) from UVA alone (in **a** and **c**) or from corresponding UVA-treated sample at 2 h or 24 h time-points (in **b**). † Significantly different (p<0.05) from cpd **2**+UVA (in **a**) or from corresponding cpd **2**+UVA-treated sample at 2 h or 24 h time-points (in **b**).

Figure S7: Lack of cytotoxicity of compounds 2 and 4 in skin cells.

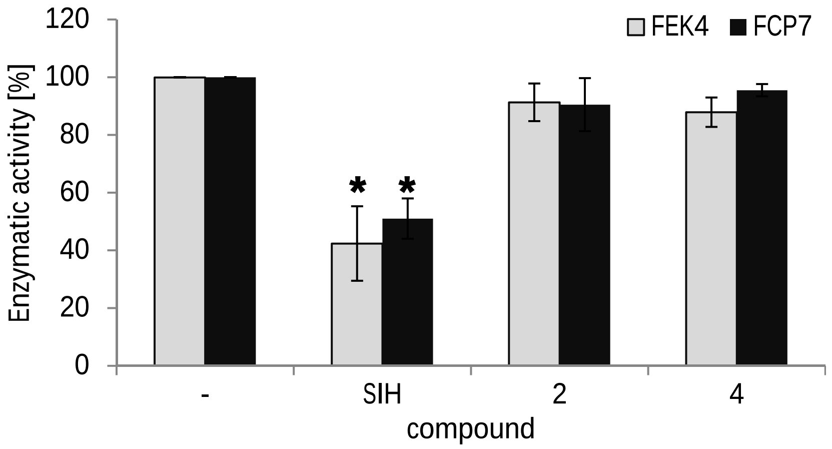

Cells were treated for 48 h with the compounds indicated at a concentration of 100 μM. Cytotoxicity was then assayed by MTT assay. Results are represented as percentage enzymatic activity of control non-treated cells and are the means +/- SD from three independent experiments. * Significantly different from corresponding non-treated control sample (p<0.05).

SUPPLEMENTARY REFERENCES

Bolte S, Cordelières FP. A guided tour into subcellular colocalization analysis in light microscopy. J Microsc 2006;224:213-32.

Dunn KW, Kamocka MM, McDonald JH. A practical guide to evaluating colocalization in biological microscopy. Am J Physiol Cell Physiol 2011;300:C723-42.

Manders EMM, Verbeek FJ, Aten JA. Measurement of co-localization of objects in dual-color confocal images. J Microsc 1993;169:375–82.
